# Medically Unexplained and Explained Physical Symptoms in the General Population: Association with Prevalent and Incident Mental Disorders

**DOI:** 10.1371/journal.pone.0123274

**Published:** 2015-04-08

**Authors:** Jonna van Eck van der Sluijs, Margreet ten Have, Cees Rijnders, Harm van Marwijk, Ron de Graaf, Christina van der Feltz-Cornelis

**Affiliations:** 1 Topclinical Centre for Body, Mind and Health, GGz Breburg, Tilburg, The Netherlands; 2 Tranzo department, Tilburg University, Tilburg, the Netherlands; 3 Netherlands Institute of Mental Health and Addiction, Utrecht, The Netherlands; 4 Department of Residency training, GGz Breburg, Tilburg, The Netherlands; 5 Centre for Primary Care, Institute of Population Health, University of Manchester, Manchester, United Kingdom; 6 Department of General Practice & Elderly Care Medicine and the EMGO+-Institute for Health and Care Research of VU University medical centre (VUmc), Amsterdam, The Netherlands

## Abstract

**Background:**

Clinical studies have shown that Medically Unexplained Symptoms (MUS) are related to common mental disorders. It is unknown how often common mental disorders occur in subjects who have explained physical symptoms (PHY), MUS or both, in the general population, what the incidence rates are, and whether there is a difference between PHY and MUS in this respect.

**Aim:**

To study the prevalence and incidence rates of mood, anxiety and substance use disorders in groups with PHY, MUS and combined MUS and PHY compared to a no-symptoms reference group in the general population.

**Method:**

Data were derived from the Netherlands Mental Health Survey and Incidence Study-2 (NEMESIS-2), a nationally representative face-to-face survey of the general population aged 18-64 years. We selected subjects with explained physical symptoms only (n=1952), with MUS only (n=177), with both MUS and PHY (n=209), and a reference group with no physical symptoms (n=4168). The assessment of common mental disorders was through the Composite International Diagnostic Interview 3.0. Multivariate logistic regression analyses were used to examine the association between group membership and the prevalence and first-incidence rates of comorbid mental disorders, adjusted for socio-demographic characteristics.

**Results:**

MUS were associated with the highest prevalence rates of mood and anxiety disorders, and combined MUS and PHY with the highest prevalence rates of substance disorder. Combined MUS and PHY were associated with a higher incidence rate of mood disorder only (OR 2.9 (95%CI:1.27,6.74)).

**Conclusion:**

In the general population, PHY, MUS and the combination of both are related to mood and anxiety disorder, but odds are highest for combined MUS and PHY in relation to substance use disorder. Combined MUS and PHY are related to a greater incidence of mood disorder. These findings warrant further research into possibilities to improve recognition and early intervention in subjects with combined MUS and PHY.

## Introduction

### Rationale

Medically Unexplained Symptoms (MUS) are highly prevalent in primary care [1–11], occupational health care [12] and specialist care [13]. They are associated with serious dysfunction such as disability in the workplace [6–8, 12, 14, 15] and high health care use [12, 14–16]. They often co-occur with common mental disorders like major depressive disorder, generalised anxiety disorder and panic disorder in primary care and in the occupational setting [12, 17, 18]; however, their specific recognition and treatment have been low [19–24]. The co-occurrence of MUS in depressive or anxiety disorders leads to a less favourable treatment response [25–31] and consequently to frequent health care use, disability and increased costs [20].

Definitions of MUS vary widely, depending on the setting [2, 5, 7, 18, 32–34]. In primary care, prevalence rates range from 1.5% to 11% depending on whether or not the criteria are restrictive [7, 35]. In general hospital settings, specific patterns of MUS are often called functional somatic syndromes, like fibromyalgia, chronic fatigue and irritable bowel syndrome, and appear to show a marked relationship with depression and anxiety [13, 36], presenting in up to 25% of patients with a depressive, anxiety or somatoform disorder in one study [5].

In the general population, a high prevalence rate (11.8%) is found for the presence of ‘any depression or anxiety disorder’ for subjects with MUS [37]. However, it is unknown to what extent the presence of MUS is a predictor of the development of depression and anxiety. Furthermore, from clinical practice, we know that comorbid substance use disorder can be an additional problem in subjects with MUS, but this has not been researched yet. Therefore, the aim of this project was to estimate prevalence and incidence rates of depression, anxiety and substance use disorder in relation to MUS in a large general population cohort, i.e. the Netherlands Mental Health Survey and Incidence Study-2 (NEMESIS-2) [38, 39].

### MUS versus explained physical symptoms

During the diagnostic medical process, physical symptoms may remain ‘unexplained’ but they may also be explained by actual physical illnesses. Escobar et al. found that both explained and unexplained physical symptoms are equally strongly associated with depression and anxiety in a cross-sectional study in a community setting [9]. However, this question has not been explored at the population level, and also, substance use disorder has not been taken into account. Furthermore, the question arises whether there is a difference between MUS and explained physical symptoms (PHY) with regard to the development of comorbid mental disorders in the long run. Additionally as a third question, these prevalence and incidence rates so far have not been explored in patients with combined explained and unexplained symptoms, although having a combination of the two might be difficult to cope with for a patient and could potentially give rise to mental disorders as well. An unfavourable course of mental disorder in the presence of physical symptoms has been described [25–27]. If individuals in the general population in the MUS, PHY or MUS+PHY groups would more often develop mental disorders over time than the control group, it would suggest that more attention should be given to the detection and treatment of these mental disorders, in order to diminish the burden of disease.

### Objectives

The objectives of this study are:

To measure prevalence and incidence rates of mood disorder, anxiety and substance use disorder in subjects with MUS compared to those with PHY or a combination of MUS and PHY.

To explore prevalence and incidence rates of comorbid mood, anxiety and substance use disorder among subjects in the general population in a control group with no explained physical symptoms and no MUS (NONE), a group with explained physical symptoms only (PHY), a group with MUS only and a group with both MUS and explained physical symptoms (MUS+PHY).

We hypothesize that the prevalence rates of mood disorder, anxiety disorder and substance use disorder are higher in the groups of respondents with MUS only, PHY only or both MUS and PHY compared to those with no physical symptoms (control respondents). We hypothesize that the effect is biggest in the combined MUS + PHY group, because of the abovementioned difficulties that arise for the patient in coping with the combination of unexplained and explained physical symptoms, and for the physician in diagnosing and managing the concomitant symptoms properly. Furthermore, we hypothesize that the same reasons apply for the incidence rates in the respective groups.

## Methods

### Design

For this study, we used data from NEMESIS-2, a nationally representative face-to-face survey held with subjects aged 18–64 at baseline, interviewed twice (once in 2007–2009 and another time in 2010–2012) with the Composite International Diagnostic Interview (CIDI) 3.0 [40]. NEMESIS-2 was approved by the Medical Ethics Review Committee for Institutions on Mental Health Care (METIGG). Respondents provided written informed consent to participate in the interview, after full written and verbal information about the study was given before and at the start of the baseline assessment.

### Setting and participants

Nationally representative population based study. As described by de Graaf et al. [38], this study was conducted as follows:

For the first wave (T_0_), in a multistage, stratified random sampling procedure, a random sample of 184 of the 443 existing municipalities was drawn. In these municipalities, a random sample of addresses of private households was drawn from postal registers. Based on the most recent birthday at first contact within the household, an individual aged 18–64 with sufficient fluency in the Dutch language was randomly selected for a face-to-face interview.

The response rate of the first wave was 65.1%. The sample was nationally representative, although younger subjects were somewhat underrepresented [38].

For the second wave (T_1_), all 6,646 participants were approached for follow-up, three years after baseline, of which 5,303 could be interviewed again (80.4% response, excluding those who were deceased).

The mean period between both interviews was 3 years and 7 days (1,102 days; sd = 64). At T_1_, there was a significantly higher chance of attrition with lower age and lower educational level. Attrition was also more likely if respondents were unemployed or born outside the Netherlands. No differences were found for gender, cohabitation status, urbanicity and having a chronic physical disorder [41].

Of the total group of 6,646 baseline respondents, 140 respondents received a shortened version of the interview, and as a consequence did not receive questions about somatic disorders. Therefore, the number of respondents in the analyses for the prevalence research question was 6,506.

### Variables

#### Definition of Medically Unexplained Symptoms

For this study, we use the following definition of MUS: *presence of one or more physical symptom(s) in the past 12 months for which no adequate organ pathology or pathophysiological basis was found*, *and for which*, *according to the subject*, *a physician was consulted and/or medication was received*, *and which caused discomfort and functional impairment in the past 4 weeks as measured by the Short Form 36 (SF-36)* [7, 33, 42, 43].

We included the presence of discomfort and functional impairment in the definition, to stay in line with the Somatoform disorders in DSM-IV [44] and the DSM-5 Somatic Symptom Disorder [32], that both require discomfort and functional impairment. SSD is ‘characterized by somatic symptoms that are either very distressing or result in significant disruption of functioning, as well as excessive and disproportionate thoughts, feelings and behaviours regarding those symptoms. The individual must be persistently symptomatic (typically at least for six months) [32].

#### Data sources and Measurement

For MUS, mental disorders and explained physical symptoms, measures were used as described in Table 1.

**Table 1:** Measures.

Measurement	Measuring instrument
**DSM-IV mental disorders**	CIDI 3.0 [45–47]
DSM-IV mood disorder (major depression, dysthymia, bipolar disorder), anxiety disorder (panic disorder, agoraphobia (without panic disorder), social phobia, specific phobia, generalised anxiety disorder) and substance use disorder (alcohol/drug abuse and dependence). Prevalence was defined as the presence of the mental disorder in the 12 months prior to the T_0_. First-incident cases of a category of disorders were defined as persons who developed a disorder in a category (mood, anxiety or substance use disorder) between T_0_ and T_1_, among those who had never experienced any separate disorder in that category at T_0_. For first time incidence in the category ‘mood disorder’ only those subjects who did not have a lifetime mood disorder before T_0_, were included in the ‘at risk’ group for this category at T_1_. Therefore, the number of respondents 'at risk' varied per group. Incidence was calculated for each separate disorder.	The interviews were conducted by professional, experienced interviewers. Clinical calibration studies conducted in various countries have found that CIDI 3.0 [40] and earlier versions [48, 49] assess anxiety, mood and substance use disorders with generally good validity compared to blinded clinical reappraisal interviews. At T_0_, a lifetime CIDI version was used. At T_1_ a CIDI version with a timeframe of the period between T_0_ and T_1_ was used.
**Explained physical symptoms**	
Respiratory disorders (asthma, chronic obstructive pulmonary disease, chronic bronchitis, emphysema), cardiovascular disorders (severe heart disease, heart attack, hypertension, stroke), stomach or intestinal ulcers, severe intestinal symptoms (only if an explanation about the cause was given such as pancreatitis, hernia abdominalis), diabetes, thyroid disorder, chronic back pain (only if an explanation about the cause was given such as neck hernia, paraplegia, caused by accident), arthritis, migraine, cancer, impaired vision or hearing.	Interview based on questionnaire of physical symptoms, in which the main physical symptoms of the CBS questionnaire can be found [50]. These physical symptoms were based on self-report by the subjects during the interview, and not by medical records [47]. Comparisons between self-reports of chronic physical disorders and medical records show moderate to good concordance [51–53]. Subjects were considered to have PHY at T_0_ if they reported to have been treated or monitored by a physician in the 12 months prior to T_0_ for one or more of the disorders, and after confirmation by two physicians, in duplicate, if symptoms should be considered to be medically explained.
**Medically unexplained physical symptoms**	
Subjects were considered to have MUS at T_0_ if their condition applied to both criteria mentioned below:	Interview based on questionnaire of physical symptoms.
1.Presence of the following physical symptoms, experienced in the past 12 months, for which the subjects indicated that they visited a physician or received medication:	All physical symptoms mentioned here (verbatim responses) were checked independently by two physicians (JES and CFC) to indicate whether or not they could be considered medically unexplained physical symptoms in general. If their judgments were not the same, they deliberated until consensus was achieved.
a) Disturbing intestinal symptoms, existing longer than 3 months, for which no indication of an explanation existed[54].	We checked the answers on the open questions to see if an explanation was given about the intestinal symptoms, such as pancreatitis or hernia abdominalis, or the back problem, such as neck hernia or paraplegia.
b) Back problems existing longer than 3 months, for which no indication of an explanation existed [55].	If this was the case, we did not include the subject in the unexplained group, but in the explained group.
c) Other illness or physical symptoms that are long lasting (open question) and unexplained:	Examples of general symptoms that we considered to be medically unexplained physical symptoms are fibromyalgia, fatigue (such as chronic fatigue syndrome), pain without medical explanation (such as stress related pain in muscles), and physical symptoms accompanied with phrases such as ‘they can’t find anything’ or ‘if only I knew’.
2. Presence of limited functioning reported in the past 4 weeks, as indicated by two or more of the following scales of the SF-36	Interview based on SF36: subscales:
a) Physical functioning: some or severe limitations in at least one of the ten items in this category	
b) Physical role functioning: any limitation reported in at least one of the four items in this category	
c) Bodily pain: pain leading to any limitation in normal work activities	
d) General health: describes mental or physical health as poor, and/or negative expectations about one's health	

### Quantitative variables and study size

#### Operationalisation of four groups

We distinguished the following groups: firstly, respondents with no MUS and no explained physical symptoms comprised the control group (NONE, n = 4168). Secondly, respondents with explained physical symptoms only, which were the physical symptoms in the checklist minus those symptoms we considered to be MUS, were defined as (PHYonly, n = 1952). Thirdly, those who had MUS, but no physical symptoms that were explained by physical disorders were grouped as MUSonly (n = 177). The final, most complex group included those who had both MUS and explained physical symptoms (MUS+PHY, n = 209).

#### Statistical methods

All analyses were performed with STATA version 11, using weighted data to correct for differences in the response rates of several socio-demographic groups (sex, age, partner status, employment situation, education) at both waves, and differences in the probability of the selection of respondents within households at baseline. Robust standard errors were calculated in order to obtain correct 95% confidence intervals and p-values [56].

For the demographic variables analysis, summary statistics were used to describe the socio-demographic characteristics of the abovementioned four groups of subjects with and without MUS: NONE, PHYonly, MUSonly and MUS+PHY.

For the analysis of prevalence rates, 12-month prevalence rates of comorbid mental disorders among these groups were calculated and multivariate logistic regression analyses were used to examine the association between group membership and the prevalence of comorbid mental disorders, adjusted for all abovementioned socio-demographic characteristics. Odds Ratios and 95% confidence intervals were presented. In the logistic regression analyses, the NONE group was used as the reference group. In additional analyses, we varied the reference group to examine the extent to which the groups with physical symptoms (PHYonly, MUSonly and MUS+PHY) differed in their odds of having and developing mental disorders.

For the analysis of incidence rates, first-incidence rates of mental disorders among these groups were calculated and multivariate logistic regression analyses were used to examine the association between group membership and incidence of comorbid mental disorders, adjusted for socio-demographic characteristics. Odds Ratios and 95% confidence intervals were presented. In the logistic regression analyses, the NONE group was used as the reference group.

#### Bias

In additional analyses, we varied the reference group to examine the extent to which the groups with physical symptoms (PHYonly, MUSonly and MUS+PHY) differed in their odds of having and developing mental disorders.

## Results

### Participants

Table 2 describes the socio-demographic characteristics of the abovementioned four groups: NONE (n = 4168), PHYonly (n = 1932), MUSonly (n = 177), MUS+PHY (n = 209).

**Table 2:** Sociodemographic characteristics of subjects with and without MUS and explained physical symptoms (N = 6,506), in unweighted numbers and weighted column percentages.

	n	NONE (n = 4168)	PHYonly (n = 1952)	MUSonly (n = 177)	MUS+PHY (n = 209)	
		%	%	%	%	p =
*Sex*						
Female	3,589	45.4	55.9	60.4	67.2	**<0.001**
*Partner status*						
With partner	4,419	65.3	71.6	65.5	73.9	**<0.001**
*Age*						
18–24	477	15.3	8.2	7.3	1.4	
25–34	1,100	23.6	11.8	18.3	7.8	
35–44	1,659	26.5	20.2	28.7	19.4	
45–54	1,559	20.4	27.6	28.1	33.1	
55–64	1,711	14.1	32.2	17.7	38.4	**<0.001**
*Employment situation*						
With paid job	4,858	80.3	71.3	65.0	50.3	**<0.001**
*Education*						
Primary, basic vocational	312	5.5	10.1	6.0	13.3	
Lower secondary	1,782	22.3	22.8	22.5	27.4	
Higher secondary	2,095	41.2	42.4	46.4	41.2	
Higher professional, university	2,317	31.0	24.7	25.2	18.1	**<0.001**

NONE: No explained physical symptoms, no MUS

PHYonly: explained physical symptoms, no MUS

MUSonly: MUS, no explained physical symptoms

MUS+PHY: both MUS and explained physical symptoms

### Descriptive data

There were significant differences between the groups regarding the following socio-demographic variables:

More women than men had physical symptoms, either explained or unexplained.

Although the majority had a partner, subjects in the MUS group were more often single.

The groups with explained symptoms had a significantly higher mean age than MUSonly, who were represented in all age groups above 24 years at similar levels.

80.3% of people in the control group had a paid job, while the employment rate in the MUS+PHY group was only 50.3%.

31.0% of the control group had a higher professional/university education, versus 18.1% in the MUS+PHY group, and around 25% in both the PHYonly and MUSonly groups.

The calculated prevalence and incidence rates were adjusted for the socio-demographic characteristics (Tables 3 and 4).

**Table 3:** 12-month prevalence of (comorbid) common mental disorders (n = 6,506).

	Any mood disorder		Any anxiety disorder		Any substance use disorder	
	%	OR (95% CI)	%	OR (95% CI)	%	OR (95% CI)
NONE	5.1	1	8.2	1	5.9	1
PHYonly	7.4	**1.59 (1.17,2.15)**	13.3	**1.80 (1.44,2.26)**	4.6	1.19 (0.78,1.82)*
MUSonly	13.5	**2.58 (1.56,4.27)**	19.0	**2.34 (1.41,3.87)**	7.1	1.66 (0.67,4.09)
MUS+PHY	10.9	**2.13 (1.25,3.63)**	17.4	**2.19 (1.46,3.29)**	8.4	3.43 (1.85,6.36)*

The analyses were adjusted for sex, age, partner status, employment situation and level of education.

NONE: No explained physical symptoms, no MUS

PHYonly: explained physical symptoms, no MUS

MUSonly: MUS, no explained physical symptoms

MUS+PHY: both MUS and explained physical symptoms

Percentages: weighted data

OR: odds ratio

95% CI: 95% confidence interval

* When PHYonly, MUSonly and MUS+PHY were respectively used as the reference group, the only significant difference was found between PHYonly and MUS+PHY for any substance use disorder.

**Table 4:** 3-year incidence of (comorbid) common mental disorders.

	Incident any mood disorder (n at risk = 4,098)		Incident any anxiety disorder (n at risk = 4,113)		Incident any substance use disorder (n at risk = 4,326)	
	%	OR (95% CI)	%	OR (95% CI)	%	OR (95% CI)
NONE	4.7	1	4.2	1	3.3	1
PHYonly	4.5	1.14 (0.72,1.80)*	5.1	1.40 (0.94,2.07)	2.4	1.14 (0.61,2.12)
MUSonly	8.7	1.89 (0.97,3.71)	5.7	1.29 (0.54,3.10)	4.0	1.76 (0.56,5.51)
MUS+PHY	10.3	2.92 (1.27,6.74)*	6.5	1.60 (0.72,3.54)	2.2	1.91 (0.54,6.77)

The analyses were adjusted for sex, age, partner status, employment situation and level of education.

NONE: No explained physical symptoms, no MUS

PHYonly: explained physical symptoms, no MUS

MUSonly: MUS, no explained physical symptoms

MUS+PHY: both MUS and explained physical symptoms

Percentages: weighted data

OR: odds ratio

95% CI: 95% confidence interval

The number at risk varies per category, because only first incidence cases were used.

* When PHYonly, MUSonly and MUS+PHY were respectively used as the reference group, the only significant difference was found between PHYonly and MUS+PHY for any incident mood disorder.

### Outcome data and main results

#### Prevalence rates

Table 3 describes the 12-month prevalence and odds ratios (ORs) of mood, anxiety and substance use disorders in the four abovementioned groups. In all groups, anxiety disorder was more common than mood and substance use disorders.

Compared to the NONE group, the MUS+PHY group showed consistently elevated ORs for the prevalence of mood disorder, anxiety disorder and substance use disorder, which mainly consisted of alcohol abuse and dependence.

Compared to the NONE group, both the PHYonly group and the MUSonly group showed significantly elevated ORs for mood disorder and anxiety disorder, but not for substance use disorder. The ORs for prevalence were the highest in the MUSonly group.

Other analyses:

When PHYonly, MUSonly and MUS+PHY were respectively used as the reference group, the only significant difference was that MUS+PHY showed a higher OR for substance use disorder when compared to PHYonly. However, this does not change the general direction of our results.

#### Incidence rates

The 3-year incidence rates and ORs of mood disorder, anxiety and substance use disorder are reported in Table 4. Because first-incidence rates were calculated, the number at risk varied per mental disorder: for mood disorder n = 4098, for anxiety disorder n = 4113, for substance use disorder n = 4326.

Compared to the NONE group, there are no significant differences in incidence rates for mood disorder, anxiety disorder or substance disorder in PHYonly and MUSonly. The only significant incidence rate occurred in the MUS+PHY group for mood disorders compared to the NONE group, OR 2.92(1.27,6.74).

Other analyses:

When PHYonly, MUSonly and MUS+PHY were respectively used as the reference group, the only significant difference was that MUS+PHY showed a higher OR for mood disorder when compared to PHYonly. However, this does not change the general direction of our results.

## Discussion

### Key results

The first main finding in this study was that our first hypothesis was confirmed. The MUS+PHY group showed an elevated prevalence of mood disorder, anxiety disorder and alcohol use disorder compared to the control group. For both PHY only and MUS only, the prevalence rate of mood disorder and anxiety disorder was significantly higher than in the control group. Compared to previous studies, this provides us with new information. Firstly, this is because our study was conducted in the general population instead of in a selected group of subjects, such as in primary care [1–8, 10–11], general hospital settings [13, 32] or in the workplace [8, 12, 14, 15]. Secondly, it is because those studies only concerned subjects with MUS and no comparison was made with subjects with PHY or combined MUS plus PHY. Thirdly, this is the first study that also takes alcohol abuse and dependence into account. The fact that the prevalence is highest in the combined group emphasizes the importance of proper diagnosis and management of this combination of symptoms and prioritizes this even above MUSonly and PHYonly. Dealing with the complexity of combined MUS and PHY seems to be difficult.

The second main finding is that our second hypothesis, which states that incidence rates would be elevated as well, and mostly in the MUS+PHY group, was confirmed for mood disorder. Thus, again, the group with combined MUS and PHY seems to be the most vulnerable of the three groups that were studied, in the long term. The incidence of mental disorders in the three groups has not been studied so far, and this finding suggests that concomitant unexplained and explained physical symptoms place the highest burden on patients and should be a specific focus of attention. This finding provides fodder for the new category in DSM-5, Somatic Symptom Disorder, that does not consider the explained or unexplained nature of the symptoms to be the crux criterion, but the distress and functional impairment that coincides with it. Future research should certainly focus on better diagnosis and treatment approaches for this patient group.

Regarding socio-demographic variables, our findings were similar to the studies in the clinical settings reporting on MUS [17], and explained physical symptoms [57]. We found that more women had physical symptoms than men, both explained and unexplained. This suggests that no particular demographic bias exists in terms of comparing findings from clinical settings to findings from the general population As would be expected, older people had more explained physical symptoms. However, all age groups above 24 years of age had only MUS to similar levels. In a recent review, comparable prevalence rates were found for MUS in a younger and middle age group, although wide ranges were reported [58]. Employment rates were the lowest in those with MUS only and those with both MUS and explained symptoms. This may be an indication of the level of dysfunction in both groups; this rate is somewhat higher than in the group with physical symptoms alone, as was previously established in a comparison between patients with rheumatoid arthritis and somatisation [49].

To our knowledge, the incidence rates of mental disorders among MUS cases as well as explained physical symptoms in the general population have not previously been studied. Our findings provide us with the opportunity to gain insight into a question that often arises in clinical practice, namely whether or not MUS precede depressive and anxiety symptoms and substance use disorder. The finding that one in every ten subjects with combined MUS and explained physical symptoms develops a mood disorder in three years time indeed suggests that subjects with the combination of explained and unexplained physical symptoms require extra attention to recognise and treat imminent mood disorders.

### Limitations

A strength of this study is that it provides new findings regarding the incidence of mood, anxiety and substance use disorder in subjects with medically explained, unexplained and combined physical symptoms.

As we used an existing database, we divided the sample retrospectively into four groups based on pre-defined clinical criteria. Although we had this limitation, we believe that our methods of operationalisation and classification are reasonable for MUS. We combined the presence of one or more medically unexplained physical symptom(s) with the presence of limited functioning, and thereby we approach essential criteria for distress and functional impairment that apply both in the DSM-IV somatoform disorders as well as the Somatic Symptom Disorder as described in the DSM 5.

### Interpretation

Our findings show a clear burden of depressive, anxiety and substance abuse or dependence disorder in the three groups of physical symptoms at the level of the general population. Our findings also show that the highest burden of disease occurs in the group of subjects with both MUS and PHY, which is the most difficult to treat. To explain to a patient that some of his or her physical symptoms can be medically explained, but other symptoms may not, can be a challenge. In view of the elevated incidence of mood disorder in this group, further research should therefore focus on treatment strategies for this specific group, with a special focus on greater attention for the development of an explanation model that both the physician and the patient can support. This can prevent the increasing insecurity and depressive symptoms that could result from the physical symptoms. Another treatment strategy could be to ensure good management of the treatment, such as paying attention to the course of the physical symptoms and regularly monitoring patients for mental problems, in a case management and disease management approach as has been suggested i.e. in the Multidisciplinary Guideline for Medically Unexplained Symptoms and Somatoform Disorders [59]. In this approach, collaboration between primary and secondary care by psychiatric consultation models, or a more elaborate model known as transmural collaborative care, is of interest [17, 60, 61]. Consultation models with the occupational health physician may be necessary as well, because of the apparent negative influence that MUS have on employment and positive outcomes in terms of Return To Work [62, 63]. However, although these models have been described and been shown to be effective in clinical research, their implementation should probably be improved. Research is needed to explore further treatment needs of these patients, as well as implementation and organizational needs of their doctors. Mental problems can also precede MUS, which is something we did not study here, but is a subject of interest for further research.

### Generalizability

An important strength of NEMESIS-2 is that it is a large nationally representative sample of the adult Dutch general population. Therefore, the results can be extrapolated to the general population of the Netherlands, and possibly to a wider area.

### Conclusions

In the general population, PHY, MUS and the combination of both are related to mood and anxiety disorders, but odds are highest for combined MUS and PHY in relation to substance use disorder. Combined MUS and PHY are related to a greater incidence of mood disorder. These findings warrant further research into possibilities to improve recognition and early intervention in subjects with combined MUS with PHY.

### Funding

NEMESIS-2 is conducted by the Netherlands Institute of Mental Health and Addiction (Trimbos Institute) in Utrecht. Financial support has been received from the Ministry of Health, Welfare and Sport, with supplementary support from the Netherlands Organization for Health Research and Development (ZonMw) and the Genetic Risk and Outcome of Psychosis (GROUP) investigators.
